# Rapid, Label-Free Prediction of Antibiotic Resistance in *Salmonella typhimurium* by Surface-Enhanced Raman Spectroscopy

**DOI:** 10.3390/ijms23031356

**Published:** 2022-01-25

**Authors:** Ping Zhang, Xi-Hao Wu, Lan Su, Hui-Qin Wang, Tai-Feng Lin, Ya-Ping Fang, Hui-Min Zhao, Wen-Jing Lu, Meng-Jia Liu, Wen-Bo Liu, Da-Wei Zheng

**Affiliations:** Faculty of Environment and Life, Beijing International Science and Technology Cooperation Base of Antivirus Drug, Beijing University of Technology, Beijing 100124, China; ahaha10010@163.com (X.-H.W.); emma_su@foxmail.com (L.S.); wanghuiqin@bjut.edu.cn (H.-Q.W.); lintaifeng@bjut.edu.cn (T.-F.L.); fangyaping777@163.com (Y.-P.F.); zhaohuimin0613@163.com (H.-M.Z.); L2384130472@163.com (W.-J.L.); liumengjia86@163.com (M.-J.L.); liuwenbo2021@163.com (W.-B.L.)

**Keywords:** antimicrobial resistance, surface enhanced Raman scattering, rapid detection, label-free, minimum inhibitory concentration, *Salmonella typhimurium*

## Abstract

The rapid identification of bacterial antibiotic susceptibility is pivotal to the rational administration of antibacterial drugs. In this study, cefotaxime (CTX)-derived resistance in *Salmonella typhimurium* (abbr. CTX^r^-*S. typhimurium*) during 3 months of exposure was rapidly recorded using a portable Raman spectrometer. The molecular changes that occurred in the drug-resistant strains were sensitively monitored in whole cells by label-free surface-enhanced Raman scattering (SERS). Various degrees of resistant strains could be accurately discriminated by applying multivariate statistical analyses to bacterial SERS profiles. Minimum inhibitory concentration (MIC) values showed a positive linear correlation with the relative Raman intensities of *I*_990_/*I*_1348_, and the R^2^ reached 0.9962. The SERS results were consistent with the data obtained by MIC assays, mutant prevention concentration (MPC) determinations, and Kirby-Bauer antibiotic susceptibility tests (K-B tests). This preliminary proof-of-concept study indicates the high potential of the SERS method to supplement the time-consuming conventional method and help alleviate the challenges of antibiotic resistance in clinical therapy.

## 1. Introduction

Since antibacterial agents were discovered in the 1920s, antibiotics have had significance in inhibiting infectious diseases and protecting human health. However, antibiotics are a double-edged sword. Excessive antibiotic abuse has led to the emergence of drug-resistant strains, which have had serious consequences worldwide [1,2]. Antimicrobial resistance (AMR) reduces the selectivity of drugs and increases the difficulty of clinical treatments, which place physical and economic burdens on patients [3]. 

In times of increasing AMR, the rapid inspection of pathogens and its AMR are crucial for determining the optimal timing of treatment and administrating antibiotics to patients. Currently, conventional methods for determining the sensitivity of bacteria to antibiotics mostly depend on measuring the changes in microbial proliferation in response to drugs. 

Such “biological assays” have inevitably taken time, ranging from days for fast-growing bacteria to weeks for slow growers [4]. Therefore, there is an urgent need to develop a novel method for the rapid identification of antibiotic-resistant pathogenic bacteria in the clinic. Raman spectroscopy is a so-called “fingerprint” spectrum that has provided a new method for the rapid detection and discrimination of bacteria in microbiological analyses [5,6,7,8]. 

Bacteria can be divided into different species or subspecies according to factors, including their colonial morphology, bacterial shape, cell wall characteristics, nutritional properties, serological reaction, physiological and biochemical styles, and genotypes. Based on these differences in the chemical structures and components of microbial cells, Raman scattering provides a whole-cell and specific vibration spectroscopic fingerprint of bacteria in a label-free and non-destructive manner, allows the rapid screening of samples, and provides an abundant ensemble of molecular vibrations. 

With the development of nanotechnology in the last two decades, surface-enhanced Raman scattering (SERS) technology achieved a sensitivity that enables Raman signals to be obtained at the single-cell or even monomolecular level [9,10,11,12,13,14]. This level of sensitivity means that collecting a small number of bacteria is sufficient to obtain reliable Raman signals [12,15]. In this way, tedious bacterial cultivation could be reduced, and the time for pathogenic bacteria analysis could be increased. Due to the improvement in intrinsic sensitivity, SERS has been successfully applied for the identification of pathogens in real samples collected from sources, including bodily fluids, drinking water, and the environment [16,17,18,19,20,21,22]. 

Recently, research areas have been further expanded to explore the metabolic changes or molecular targets in bacterial cells exposed to antibiotics, heavy metals, and other drugs using Raman technology [23,24,25,26,27,28,29,30,31]. Some researchers have proposed using the concepts of a “Raman phenotype,” “Raman profile,” or “Ramanome” combined with multivariate statistical analyses to distinguish drug-resistant and sensitive bacteria based on the whole-cell responses to drugs [26,27,28,29,32,33,34,35,36].

Acquiring resistance in a bacterial population is a relatively long procedure in which bacterial cells are gradually mutated, and the structures and molecules are changed. Finally, resistant cells develop in the dominant population exposed to drugs. In the induction procedure, it remains unclear whether these dynamic structural alterations can be rapidly reflected on the Raman spectrum. Addressing this question will provide a means to rapidly identify the drug-resistant bacteria, predict the resistant trends, and elucidate the mechanisms underlying the resistance. 

For this purpose, we applied a portable Raman spectrometer to dynamically monitor the population of *Salmonella typhimurium* with or without antibiotics and rapidly identified their antibiotic-resistant characteristics. Therefore, cefotaxime (CTX)-susceptible *S. typhimurium* ATCC 14028 strains (abbr. CTX^s^-*S. typhimurium*) were co-cultivated with CTX, a commonly used antibiotic in the clinic, to mutate and screen CTX-resistant strains (abbr. CTX^r^-*S. typhimurium*) for 3 months. 

SERS technology has been used to accompany screening processes to rapidly and dynamically monitor the differences between antibiotic-resistant strains and susceptible strains in label-free manners. Subsequently, the vibration changes reflected in the Raman profiles were analyzed to explore the molecular targets of antibiotic action. Finally, SERS methodology was approved by identifying the results collected by Kirby–Bauer antibiotic susceptibility tests (K-B tests), minimum inhibitory concentration (MIC) determinations, and mutant prevention concentration (MPC) tests. 

The results showed that the sample preparation and SERS collection were finished in a few hours, which indicated its rapid diagnostic ability. The antibiotic-resistant strains could be easily and accurately discriminated with susceptible strains using the SERS method combined with multivariate statistical analyses. It showed high sensitivity and accuracy in the identification of drug-resistant bacteria, investigation of the drug effects on bacteria, and exploration of the antibiotic mechanisms. This research can help mitigate the rising challenges of antimicrobial resistance and excessive misuse of antibiotics.

## 2. Results and Discussion

### 2.1. Mutation and Identification of Drug-Resistant Strains

**Figure 1:**
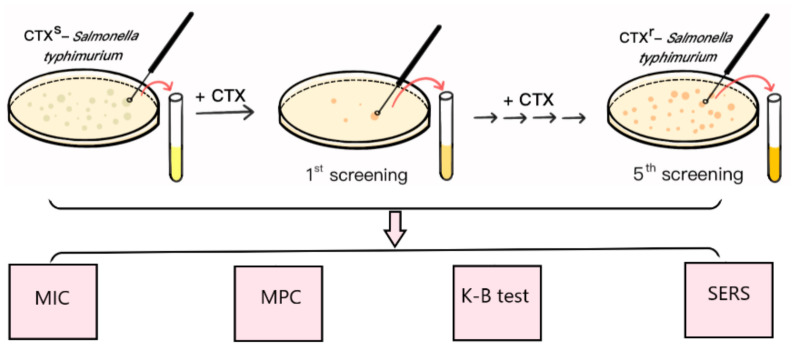
Schematic diagram of mutant induction and SERS detection.

*S. typhimurium* ATCC 14028 is a quality control strain that is commonly used in antimicrobial susceptibility tests. CTX is a third-generation cephalosporin that is widely used to treat Gram-negative bacterial infections in the clinic. In this study, the multidrug resistant *S.* *typhimurium* strain (CTX^r^-*S. typhimurium*) was the fifth round of mutants derived from *S. typhimurium* ATCC 14028 after step-by-step exposure to incremental amounts of CTX (Figure 1).

Initially, the CTX MIC of *S. typhimurium* ATCC 14028 was 0.5 μg⋅mL^−1^, which was lower than the interpretation standard (1 μg⋅mL^−1^) of the drug sensitivity test results, indicating the susceptible characteristics of the strain. The CTX MPC of sensitive *S. typhimurium* was 2 μg⋅mL^−1^ because there was no bacterial growth on the plates as the concentration of CTX reached 2 μg⋅mL^−1^ in MPC tests. To induce the antibiotic-resistant strains, the initial screening concentration of CTX-containing plates was set at 1 μg⋅mL^−1^, which was half the MPC value of the susceptible strains. 

At this concentration, most of the susceptible strains were killed by CTX, and only a small portion of bacterial cells survived and “mutated” to resist antimicrobial drugs. The surviving single clones formed on the drug-containing plates were selected and cultivated overnight in drug-free liquid MHB medium to recovery bacterial activities, followed by the MIC assay, K-B test, and SERS method to verify the increasing resistant characteristics; this was the first generation of screening (Figure 1). 

After the fifth round of mutation, CTX MIC gradually increased from 0.5 μg⋅mL^−1^ to 4 μg⋅mL^−1^ after 3 months. The results of antibiotic susceptibility tests showed that the diameter of the inhibition zone of CTX was gradually reduced from 27.3 mm to 14.1 mm (Supplementary Table S1). Furthermore, the K-B test clearly showed that the selected resistant strains were not only resistant to CTX but were also resistant to other types of antibiotics, such as cefoxitin, cefazolin, cefuroxime (cephalosporins), streptomycin (aminoglycoside antibiotics), and ciprofloxacin (4-quinolones antibacterial agents) (Supplementary Table S1). 

Subsequently, *S. typhimurium* gradually enhanced antibiotic resistance by increasing the amount of CTX after the fifth round of mutation. Under the action of CTX, the resistant strain became the dominant organism in the bacterial population and finally acquired multidrug resistance after 3 months. This strain was called the multidrug resistant *S. typhimurium* strain (referred to as CTX^r^-*S. typhimurium* for convenience).

### 2.2. Rapid Detection and Discrimination of CTX^s^-S. typhimurium and CTX^r^-S. typhimurium by SERS

**Figure 2:**
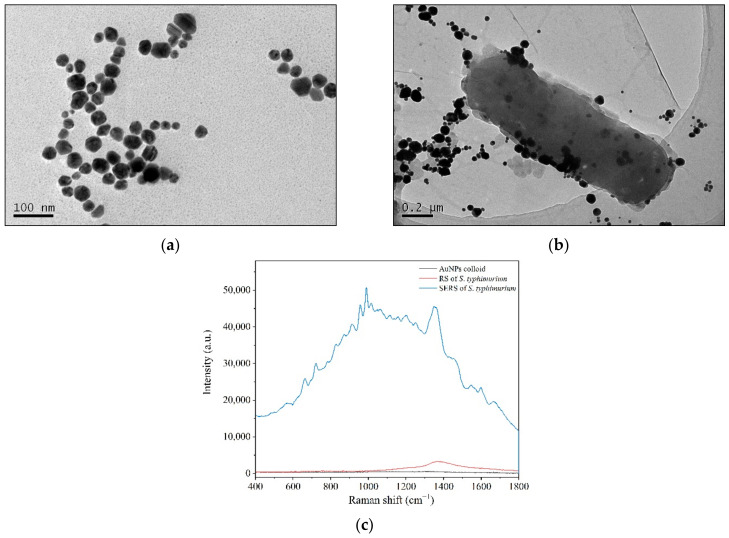
Enhanced bacterial Raman signals by directly applying colloidal AuNPs to bacterial cells. (**a**) TEM of AuNPs; (**b**) AuNP aggregating and adhering to bacterial cell surfaces under TEM; (**c**) RS and SERS of *S. typhimurium*.

Aside from the above biological assays, SERS technology has been applied to record the spectral differences associated with the screening process. Colloidal Au nanoparticles (AuNPs) were used as an enhanced substrate and were red wine in color with a maximum absorption of approximately 532 nm. AuNPs were nearly spherical with particle sizes mainly ranging from 40 to 60 nm in TEM images (Figure 2a). The AuNPs showed good dispersion characteristics and could be stored for at least 3 months without precipitation [37]. 

When colloidal AuNPs were applied to the bacterial solution, the nanostructured Au particles gathered around the bacterial cells, and some were adsorbed onto the bacterial cell surfaces to facilitate the surface-enhanced effects (Figure 2b). The Raman peaks of *S. typhimurium* clearly and quickly showed up in the SERS spectra (Figure 2c), and the data could be collected in several seconds. Although the enhanced mechanisms of AuNPs are not well understood, it is well known that the enhanced Raman signals mainly arise from the “hot spots” that form by the aggregation of gold nanoparticles [38,39]. 

From the TEM images, parts of AuNPs gathered and adsorbed on the surfaces of bacterium that formed the “hot spots” (Figure 2b). Bacteria are several orders of magnitude larger than nanoparticles. There are many biological macromolecules, such as proteins, on the cell surface. The gathering of gold particles on the surface of bacteria is partly due to the electrostatic adsorption between the negatively charged AuNPs and the positively charged proteins. 

Therefore, AuNPs adhered to some areas of cell surface far more than to others and did not wrap around the bacteria in a uniform way (Figure 2b). In addition, a majority of biological molecules are Raman active. In an acidic environment, it is difficult to maintain the integrity of bacterial cells. In this way, the bacterial SERS signals may be generated by cell surfaces and the leakages showing the signatures of both cell surfaces and internal molecules. 

Thus, in a complex system, such as a bacterial cell, SERS signals carry information about the mixture of inherent biomolecules [40,41,42,43], which in turn gives birth to a whole-fingerprint spectrum of a whole cell. Therefore, as a laser beam penetrated through a sample of bacteria and colloidal AuNPs, it could produce an identifiable Raman spectrum with high sensitivity to provide information based on whole-cell molecular vibrations.

**Figure 3:**
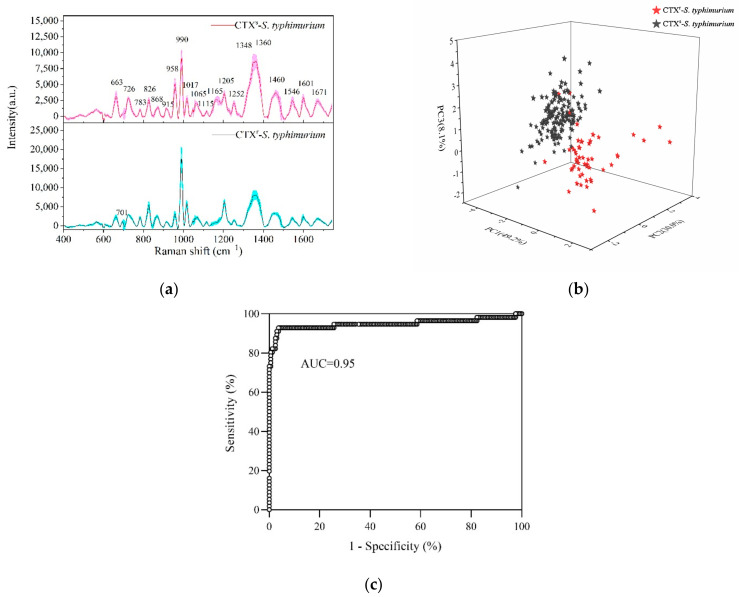
Detection and discrimination of CTX-susceptible *S. typhimurium* (CTX^s^-*S. typhimurium*) and multidrug-resistant *S. typhimurium* (CTX^r^-*S. typhimurium*) by SERS. (**a**) Average SERS spectra of CTX^s^-*S. typhimurium* (red line) and CTX^r^-*S. typhimurium* (black line) with the standard deviation (colorful area). (**b**) PCA plot of PC1 (49.2%), PC2 (30.0%), and PC3 (8.1%) showing CTX^s^-*S. typhimurium* strains (black stars) separated with CTX^r^-*S. typhimurium* strains (red stars) at 95% accuracy. (**c**) Area under ROC curve (AUC) reached 0.95 based on cross validation for a PCA-LDA model by bacterial SERS detection.

As shown in Figure 3a, the Raman fingerprints of susceptible and resistant *S. typhimurium* were mainly distributed in the 600–1700 cm^−1^ region with the standard deviations of mean SERS spectra not exceeding 10%. CTX^s^-*S. typhimurium* and CTX^r^-*S. typhimurium* showed most of the typical Raman peaks in the same position. The strong Raman peaks included 990, 1348, 1360, 1460, 1205, 958, 1017, 663, and 826 cm^−1^. The other Raman peaks of *S. typhimurium* and its tentative assignments are shown in Table 1. 

Comparing the drug-resistant strains with susceptible strains, the typical characteristic was a Raman peak at 1165 cm^−1^ that was significantly decreased or even disappeared. This peak was associated with C-C and C-N stretching vibrations in proteins [5,8]. Furthermore, CTX^r^-*S. typhimurium* strains showed more differences with CTX^s^-*S. typhimurium* strains in the 700–1300 cm^−1^ region. For example, the peak intensity at 958 cm^−1^ in drug-resistant strains was significantly decreased compared to susceptible strains, whereas the peaks at 990, 1017, 826, and 701 cm^−1^ were increased dramatically. 

Generally, the Raman peak intensity is related to the content of an analyte in samples. The decreased or even absent peak at 1165 cm^−1^ indicated that the protein contents in the bacterial cell were decreased under the effect of CTX. Cephalosporins bind to penicillin-binding proteins to facilitate the bactericidal effects on bacterial cells. In turn, this complex inhibits the transpeptidation action on cell wall synthesis, leading to bacterial lysis or even cell death [57]. 

During this course of action, CTX hinders protein synthesis in cell walls and changes the surface structures of bacteria [57]. These changes are directly reflected in the SERS spectra wherever the protein-related Raman peaks clearly decline. Therefore, the changes in SERS fingerprints provide insight into the specific interaction sites of relevant drugs in bacterial cells.

As most of the typical peaks in susceptible and resistant strains were in the same positions, it was difficult to directly distinguish them from Raman spectra alone. Therefore, it was necessary to use multivariate statistical analyses for help in discriminating. PCA was used to reduce the dimensionality of data (data compression) and then to extract the main characteristic components from the original data (data interpretation) [41]. LDA was used to determine the discriminant function that maximizes the variance between different groups and minimizes the variance within a group. 

PC-LDA reduces the quantity of data and improves the accuracy of discrimination effectively. By using a PC-LDA model, one can quickly and accurately classify and identify unknown samples. The Raman data at 600–1400 cm^−1^ were selected to process the PCA and LDA. The PCA plot showed that the CTX^s^-*S. typhimurium* strains were accurately separated with the CTX^r^-*S. typhimurium* strains in which PC1 (49.2%), PC2 (30.0%), and PC3 (8.1%) explained 87.3% of the accumulative variance contribution (Figure 3b). 

Leave-one-out cross validation was used for the PCA-LDA model, and the accuracy of the model reached 95% according to the receiver operating characteristic (ROC) curve analysis (Figure 3c). Compared with the common biological assay, which takes at least 1–2 days to provide validated results, SRES technology only needs several hours to report bacterial-resistant characteristics, indicating its rapid, accurate, and sensitive detection ability.

### 2.3. Dynamically Monitoring the Increasing Drug Resistance of S. typhimurium by SERS

**Figure 4:**
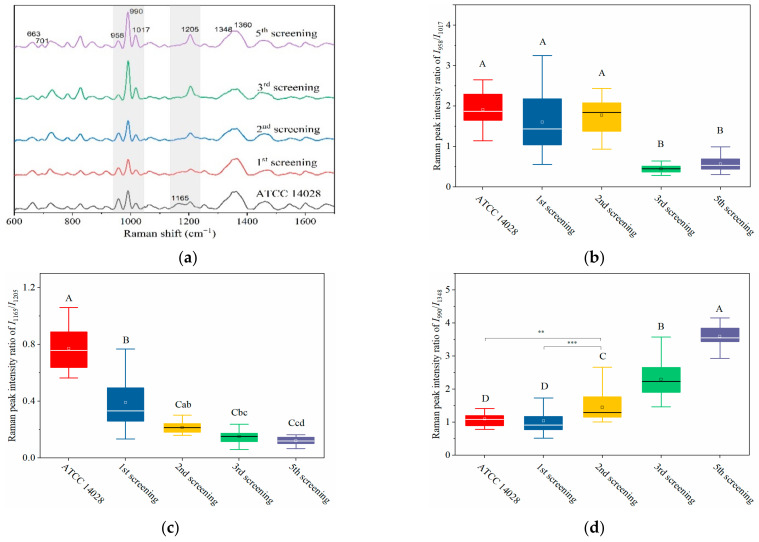

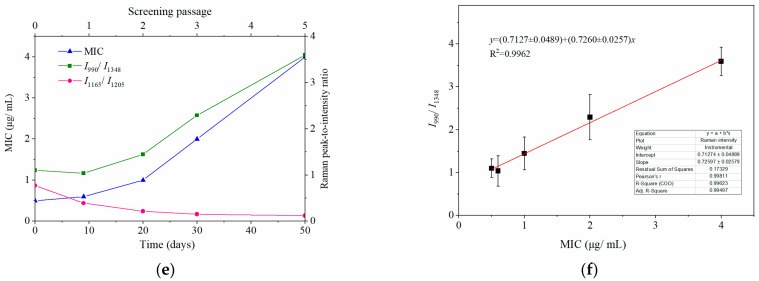
Dynamically and rapidly monitoring the increasing drug resistance of *S. typhimurium* by SERS. Dynamic changes in SERS spectra with different screening passages (**a**). Comparing the relative Raman peak intensities of *I*_958_/*I*_1017_ (**b**), *I*_1165_/*I*_1205_ (**c**), and *I*_990_/*I*_1348_ (**d**) with the screening process. (**e**) The relationship between MIC and Raman peak-intensity ratios of *I*_990_/*I*_1348_ and *I*_1165_/*I*_1205_ during the mutation procedure. (**f**) Linear relationship between MIC values and the relative Raman peak intensities of *I*_990_/*I*_1348_ indicated that the MIC could be predicted according to the typical Raman peak intensity. Note: the capital letters represent the significant differences at *p* < 0.0001, *** means *p* < 0.001, ** means *p* < 0.01, and the lowercase letters show *p* < 0.05.

Based on its sensitivity, SERS technology has been utilized to predict the resistance tendency of *S. typhimurium* in the process of acquired drug resistance. As shown in Figure 4a, the SERS spectra of mutant strains showed dynamic and regular changes with different rounds of screening. In general, with the increasing amount of CTX, typical Raman peak intensities at 1165, 958, and 663 cm^−1^ gradually presented with decreasing trends, whereas 701, 990, 1017, and 1205 cm^−1^ were steadily increasing (Figure 4a). 

Particularly in the 950–1030 cm^−1^ and 1130–1225 cm^−1^ regions (marked in grey shadows in Figure 4a), *I*_958_/*I*_1017_ and *I*_1165_/*I*_1205_ showed a declining tendency with the increase of screening algebra (Figure 4b,c). From the third round of screening, the ratios of *I*_958_/*I*_1017_ showed a significant difference compared to the first two rounds of screening (*p* < 0.0001; Figure 4b).

It was interesting to observe that the MIC of bacteria from the third round of screening reached 2 μg mL^−1^, indicating that the susceptible *S. typhimurium* was mutated to resist CTX (Figure 4e). It is possible that the changes in *I*_958_/*I*_1017_ reflected the leap from quantitative to qualitative changes. This “action site” had the threshold effect and could be used for the qualitative analysis of CTX^r^-*S. typhimurium*. 

The other pair of Raman peaks was 1165 and 1205 cm^−1^ (*I*_1165_/*I*_1205_), which showed the same variation trend as *I*_958_/*I*_1017_ (i.e., intensity of 1205 cm^−1^ steadily increased, and 1165 cm^−1^ decreased or even disappeared). The difference between *I*_1165_/*I*_1205_ and *I*_958_/*I*_1017_ was that the sites at *I*_1165_/*I*_1205_ were more sensitive to CTX, because the *I*_1165_/*I*_1205_ declined rapidly as bacterial cells were exposed to CTX (Figure 4c). These results indicate that the subtle changes in bacterial cells can be sensitively captured by SERS as bacterium exposure to antibiotics.

Another prominent symbol of increasing drug resistance was the steadily increasing intensity of *I*_990_/*I*_1348_, in which intensities of 990 cm^−1^ significantly increased from the second round of screening, whereas 1348 cm^−1^ remained basically unchanged (Figure 4a,d). This suggest that bacteria might synthesize new proteins to resist CTX in a gradual accumulation process. The intensity of *I*_990_/*I*_1348_ showed a positive correlation with the MIC values, and the R^2^ reached 0.9962 (Figure 4e,f). Thus, the bacterial resistance and its degree of resistance (MIC) could be rapidly predicted according to their Raman phenotypes and typical Raman peak intensities.

It is worth noting that the changes in bacterial concentrations also cause variation in Raman peak intensities. Therefore, to eliminate these possible interferences, it was necessary to ensure the consistency of bacterial density in the SERS tests. To this end, the OD_600_ of the bacterial suspension was equal to 0.25 for the SERS test. Therefore, the different bacterial Raman phenotypes can be used to rapidly identify drug-sensitive or resistant characteristics, and a classification model can be established in light of these spectral changes.

### 2.4. PCA-LDA for the Identification of Different Degrees of Drug-Resistant Strains

**Figure 5:**
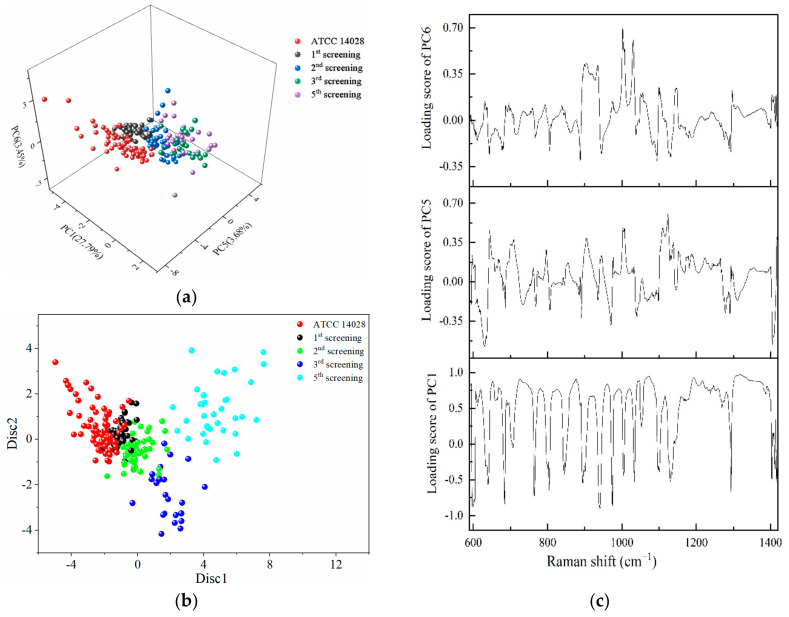
PCA and LDA results for the identification of different degrees of drug-resistant *S. typhimurium* strains. (**a**) The three-dimensional scatter plots of PC1 (29.79%), PC5 (3.68%), and PC6 (3.45%) for the spectral range of 600–1400 cm^−1^ obtained from different screening passages of *S. typhimurium* under the action of CTX. (**b**) Scatter plot of PC-LDA results in which the discriminant functions 1 and 2 as the abscissa and ordinate. (**c**) Loading plots of PC1, PC5, and PC6.

The regions of 600–1400 cm^−1^ were selected to conduct PCA, and the first six principal components contributing to 67.6% of the accumulative variance contribution were input for LDA analyses. From the three-dimensional scatter plot in Figure 5a, PC1 (29.79%), PC5 (3.68%), and PC6 (3.45%) were selected to build a coordinate system, in which different rounds of screening bacteria were concentrated in different regions. The susceptible strains and different degrees of mutated strains were arranged in a consecutive order along the PC5 axis.

The dots representing the susceptible strains were very distant from the fifth screening strains. The dots on the first, second, and third round of mutations were arranged sequentially between the susceptible strains and multidrug-resistant strains. The longer distances were further implied with the increasing variations gradually occurring in a serial generation of bacterial cells under the selected pressure of antibiotics. Figure 5b shows the scatter plot of PC-LDA in which the discriminant functions 1 and 2 as the abscissa and ordinate showed the same tendency as in Figure 5a. 

According to the loading plots in Figure 5c, a higher loading score indicates that more representative Raman peaks can show the differences between the susceptible and resistant cells. Therefore, a loading value above 0.8 was selected, and the wavebands obtained were 679–684, 719–754, 866–881, 938–940, 951–965, 978–998, 1017, 1040–1088, 1162–1196, 1200–1230, 1242–1247, and 1300–1379 cm^−1^. These results obtained by PCA further validate the results shown in Figure 3a.

The PC-LDA model was utilized to identify and discriminate of drug-susceptible strains with different degrees of drug-resistant strains during the screening process. The accuracy of the PC-LDA model reached 86.8% using leave-one-out cross validation. The SERS results were consistent with the results collected by the MIC, MPC, and K-B tests. 

The data shown in Table 2 further indicated that the mutation was a steadily accumulating process. The emergence of drug resistance in bacteria is a natural process that can be accelerated by exposure to antibiotics [57]. Under the action of antibiotics, the drug-sensitive strains were gradually eliminated, and the resistant bacteria became the dominant population with the screening action and reproduction procedure. The tolerant or resistant bacteria increased with the concentration and reaction times of the treated drugs (Figure 1, Table 2).

The period of bacterial mutation varied with different kinds of drugs and different species of bacterial cells [2]. Some bacteria quickly became resistant to drugs, and some became resistant more slowly. We found that *Staphylococcus aureus* could develop drug resistance after several days of exposure to oxacillin (data not published). Otherwise, *S. typhimurium* needed more time to produce resistance to CTX. The Raman peak intensity of *S. aureus* and *Escherichia coli* decrease rapidly within 2 h of exposure to antibiotics [25,46], which is consistent with the conclusion of our study. 

After the withdrawal of CTX, bacteria were transferred into complete culture medium to restore cell vitality. However, the changes that occurred in bacterial cells could still be detected by SERS, indicating that bacteria quickly begin the mechanism to resist drugs as soon as they contact it. This level of sensitivity cannot be reached by the conventional method. Thus, this study furthermore demonstrates that SERS can be applied as an important means to not only study the interaction between antibiotics and bacteria but also provide an effective method for the rapid identification of drug-resistant strains.

## 3. Materials and Methods

### 3.1. Bacteria and Antibiotics

The CTX-susceptible *Salmonella typhimurium* ATCC 14028 strain (abbr. CTX^s^-*S. typhimurium*) was collected from the China Center of Industrial Culture Collection (Beijing, China). The multidrug resistant *S. typhimurium* strain (abbr. CTX^r^-*S. typhimurium*) is a mutant derived from CTX^s^-*S. typhimurium* after repeated exposure to CTX (Sigma-Aldrich, St, Louis, MO, USA) for 3 months. Mueller-Hinton broth (MHB) and Mueller-Hinton agar (MHA) culture medium were purchased from Beijing Land Bridge Technology Co., Ltd. (Beijing, China). CTX-containing plates were freshly prepared with MHA medium. Bacterial samples were conserved in MHB culture medium containing 20% glycerol and stored at −80 °C.

The CTX sodium stock solution was filtered through a 0.22 μm sterilization filter (Millex-GS, Millpore) and stored at −20 °C. Oxoid CTX antimicrobial susceptibility disks (30 μg mL^−1^) were purchased from Thermo Fisher Scientific Inc. (Walsham, MA, USA). Oxacillin (1 μg per piece), cefazolin (30 μg per piece), latamoxef (30 μg per piece) and other antibiotics were bought from Hangzhou Microbial reagent Co., Ltd. (Hangzhou, China). Chloroauric acid and sodium citrate were purchased from the Sinopharm Chemical Reagent Co., Ltd. (Beijing, China).

### 3.2. Mutation and Screening of Drug-Resistant Strains

The mutation was generated by co-culturing *S. typhimurium* ATCC 14028 bacterial cells with CTX in MHA plates at 37 °C. The concentration of CTX used in the mutation process was verified with the increasing generation of mutations but was below the MPC. First, *S. typhimurium* ATCC 14028 at a concentration of 1.5 × 10^9^ CFU mL^−1^ was prepared in advance. 

An appropriate 50 μL of bacterial suspension was spread on CTX-containing plates and cultured at 37 °C for about 48 h. Most of the susceptible *S. typhimurium* strains were killed with CTX, and only a small portion of strains could survive and grow into colonies. Single large colonies were picked up and re-cultivated in liquid MHA medium without any drugs to recover the bacterial activities. These identified strains were considered the first generation of screening. 

The above procedures were repeated to enhance the acquired antibiotic resistance of *S. typhimurium*, and the selected strains were considered the next generation of screening. The drug concentrations used in the mutation experiment were based on the results of MIC and MPC tests. As mutations were generated, at least five monocolonies were picked up, re-cultivated, identified, and stored separately for the following studies. The mutated characteristics of selected colonies were identified by MIC and MPC assays, the K-B test, and the SERS method.

### 3.3. Identification of Drug-Resistant Strains

Antibiotic susceptibility tests were carried out to different generations of the survival strains by using the K-B test recommended by the National Committee for Clinical Laboratory Standards (NCCLS). The MIC of CTX for each bacterial strain was determined by the constant broth dilution method inoculated with 5 × 10^5^ CFU mL^−1^ and incubated at 37 °C without agitation [4]. A serial of CTX in the tubes was set as 32, 16, 8, 4, 2, 1, 0.5, and 0.25 μg mL^−1^. 

The MIC corresponded to the lowest concentration of CTX that completely inhibited bacterial growth after 18 h of exposure. The minimal CTX concentration that allows no mutant recovery when approximately 10^10^ cells are applied to CTX-containing agar is defined as the MPC. The MPC was measured by adding bacterial solution to CTX-containing MHA medium, followed by cultivation at 37 °C for about 96 h. The CTX concentration differed depending on the bacterial cell (wild-type and resistant mutants) and MIC values.

### 3.4. SERS Test and Data Processing

Colloidal gold nanoparticles (AuNPs) used as an enhanced substrate were synthesized in our laboratory according to the method by Wei et al. [37]. A portable Raman spectrometer (RamTracer-200, OptoTrace Technologies, Inc., Suzhou, China) was applied to collect the bacterial Raman signals, which were 20 × 18 × 10 cm in size. The bacterial suspension for the SERS test was prepared by cultivating in liquid MHB medium to the logarithmic growth stage. The susceptible cells were harvested after 6 and 12 h of culturing for mutations. 

The culture medium was centrifuged at 8000 rpm for 10 min at 4 °C, and the supernatant was discarded. Then the bacterial cells were washed three times with 0.75% NaCl solution and adjusted to a cell concentration an optical density (OD_600_ = 0.25) value that corresponds to ~3 × 10^8^ CFU mL^−1^, as measured by an ultraviolet spectrophotometer (UH4150, Hitachi, Tokyo, Japan). A volume of 0.1 mL of bacterial solution was mixed with 0.5 mL colloidal AuNPs in a glass vial. The test was conducted using a 785 nm laser excitation light and 200 mW of laser power. 

Each spectrum had an integration time of 20 s, and was scanned three times. The spectral range was from 400 to 1800 cm^−1^, and the resolution was 4 cm^−1^. In each mutation step, one bacterial strain was collected from at least five Raman spectra, and each generation of screening contained at least six bacterial strains in parallel. To reduce interference from the fluorescence background and instrument noise, the original spectra needed to be pre-processed by baseline correction, normalization, and smoothing using OptoTrace proprietary software (OptoTrace Technologies, Inc., Suzhou, China). 

For baseline correction, a second-order polynomial was fitted to the range of 400–1800 cm^−1^ and subtracted from the spectra. The normalization was conducted by setting the intensity of the internal standard peak to 1000. PCA and LDA were statistically analyzed using SPSS Statistics software (v. 26). Leave-one-out cross validation was applied to verify the PCA-LDA model. One-way analysis of variance was used to compare the differences among various groups by GraphPad Prism (version 8.0). The figures were plotted with Origin software (v. 8.0).

### 3.5. Transmission Electron Microscopy of the Combination of AuNPs and Bacterial Cells

The combination of AuNPs and bacterial cells was analyzed by transmission electron microscopy (TEM, JEM-1200EX, JEOL, Akishima, Japan). The Au colloid was mixed with the bacterial suspension (3 × 10^8^ CFU mL^−1^) at a 5:1 ratio (*v:v*), and the mixture was diluted 100 times with ultra-pure water. Then, 10 μL of dilution was added to a carbon support film and naturally dried in a dustless environment. TEM measurements were made at an acceleration voltage of 120 KV and 0.14 nm resolution.

## 4. Conclusions

In this study, a portable Raman spectrometer was applied with commonly regular biological assays to rapidly record the acquired drug resistance of *S. typhimurium* under the action of CTX. The results showed that drug-susceptible *S. typhimurium* strains gradually obtained drug resistance after five rounds of exposure and screening in 3 months. The mutant strains finally became multidrug-resistant with exposure to more than three different kinds of antibiotics in the K-B tests. 

CTX^r^- and CTX^s^-*S. typhimurium* strains showed almost the same Raman peaks in their SERS spectra; however, several peak intensities were significantly different. In particular, the 950–1225 cm^−1^ region displayed a dynamic and regular changes with different rounds of screening in the mutant strains. When the characteristic bands were selected for multivariate statistical analyses, the drug-resistant strains were accurately identified with the susceptible strains with an accuracy that reached 95%. 

The SERS results were rapidly reported in several seconds and were consistent with the results from the MIC and antimicrobial susceptibility tests. The data indicated that using portable equipment with the SERS method could be a supplementary means to a time-consuming method to help accurately identify drug-resistant strains on-site in the clinic.

## Figures and Tables

**Table 1:** Tentative assignments of the typical Raman peaks of CTX^s^-*S. typhimurium* and CTX^r^-*S. typhimurium* strains.

Raman Shift (cm^−1^)	Peak Assignments
663	*δ*(G) [5,18,44,45,46], *υ*(C-S) in Cys [5,8,44,45,47]
701	τ(C-C) in Tyr [45], Cholesterol, cholesterol ester [48]
726	A [5,8,18,44,46], *N*-acetyl-d-glucosamine (cell wall) [47], CoA/acetyl-CoA [18], C-S (protein), *ρ*(CH_2_) [49]
783	*υ*(PO_2_^−^) [5,45,50], C, A ring breathing [5,8,18,45,46,50,51]
826	*υ*(PO_2_^-^) [5,50], nucleic acids [8,50,52], Tyr [8], υ(C-C) in 1,4 glycosidic link [45]
868	*υ*(CN), υ^s^(CON), *δ*(CCH) aliphatic [44], *υ*(CC) [47,53], υ^s^(lipid) [5,8,44], υ(COC) [45,53], ribose [45]
915	*υ*(C-C) of Pro [50], Glucose, ribose vibration [54], Deoxyribose [18], C-O, C-OH, *υ*(C-COO^−^) (carbohydrates) [53]
958	*δ*(C=C) [44], *υ*(C-N) [18,45], *υ*(C-O) [18],
990	Phe [44,47], β-sheet [8]
1017	Phe [44]
1048	Carbohydrates [18,47], C-O [18,47], *δ*(C-OH) [18], polysaccharide [45]
1065	Phospholipids [18], Phe [55], fatty acid [48]
1115	Tyr, *δ*(NH_3_^+^) [44], δ(CH_2,6_) and C_1_-C_α_-H_α_ bend [56]
1165	*υ*(C-C)/*υ*(C-N) of protein [5,8]
1205	Tyr [8,18], Phe, Try [5,8,18] (protein), *υ*(CN) [57], *δ*(H-C-C) [57]
1252	Amide II [5,8,18,44,48,50], *υ*^as^(PO_2_^−^) [44,46], lipids [18], G, C (NH_2_) [58]
1348	A [5,8,46], G [5,8], Try [18,44]; *δ*(CH) [5,8,44,53], Amide III [48]
1360	*δ*(CH_2_) [44], Try [18,44]
1460	Amide II, *δ*(CH) (protein, DNA/RNA, lipid, carbohydrate) [5,8,18,44,47,53]
1546	*υ*^as^(NO_2_) [8,44], *υ*(CH_2_), exopolysaccharide [44], *υ*(C=C) [53], Amide II [45,53]
1601	Amide I [18,50], Tyr [5,8,44,45]
1671	Amide I [5,8,44,45,53,55,59], *ν*(C=C) [60]

Note: A, adenine; G, guanine; T, Thymine; C, Cytosine; U, Uracil; Phe, phenylalanine; Tyr, Tyrosine; Try, tryptophan; Cys, cysteine; Pro, proline; hydroxyproline; CoA, Coenzyme A; *δ*, deformation; *ν*, stretching; *τ*, twisting; *ρ*, rocking; *δ*, in plane bending; *γ*, out of plane bending; and *ω*, wagging.

**Table 2:** The application of leave-one-out validation to PC-LDA model to predict different rounds of screening strains.

Screening Algebra		Prediction Group				Total	Sensitivity (%)	Specificity (%)
	ATCC 14028	1st Screening	2nd Screening	3rd Screening	5th Screening			
ATCC 14028	66	3	6	0	0	75	88	96.1
1st screening	6	40	6	0	0	52	77	98.3
2nd screening	0	0	40	3	1	44	91	92.4
3rd screening	0	0	2	19	0	21	90.5	97.1
5th screening	0	0	0	3	33	36	91.7	99.5

## Supplementary Materials

The following are available online at https://www.mdpi.com/article/10.3390/ijms23031356/s1.
