# Supplementary material for: Rapid, Label-Free Prediction of Antibiotic Resistance in Salmonella typhimurium by Surface-Enhanced Raman Spectroscopy
Source: Int J Mol Sci. 2022 Jan 25;23(3):1356. doi: 10.3390/ijms23031356 (PMC8835768; doi:10.3390/ijms23031356)
Supplement: Supplementary file 1 [file ijms-23-01356-s001.zip › ijms-1482178-supplementary.pdf]

International Journal of *Molecular Sciences*

MDPI

Supplementary Table S1. Antimicrobial susceptibility tests of CTX^s-*S. typhimurium* and CTX^r-*S. typhimurium*. (Unit: mm).

Antibiotics	CTX^s-*S. typhimurium*		CTX^r-*S. typhimurium*		Criteria for interpretation		
	Inhibition diameters	Results	Inhibition diameters	Results	S	I	R
Cefotaxime	27.3	S	14.1	R	≥26	23-25	≤22
Cefoxitin	23.9	S	13.0	R	≥18	15-17	≤14
Cefazolin	23.0	S	13.3	R	≥23	20-22	≤19
Cefradine	8.0	R	7.4	R	≥18	15-17	≤14
Latamoxef	25.9	S	16.8	I	≥23	15-22	≤14
Cefuroxim	21.0	S	7.9	R	≥18	15-17	≤14
Erythromycin	7.5	R	8.6	R	≥23	14-22	≤13
Streptomycin	11.5	I	9.0	R	≥15	12-14	≤10
Novobiocin	7.5	R	7.0	R	≥17	14-16	≤13
Ciprofloxacin	33.9	S	14.8	R	≥31	21-30	≤20
Ampicillin	9.5	R	7.5	R	≥17	14-16	≤13
Piperacillin/Tazobactam	22.8	S	12.0	R	≥21	18-20	≤17
Meropenem	8.0	R	7.5	R	≥23	14-15	≤19

Note: S, sensitive; I, intermediary; R, resistant.
